# A systematic review of the prevalence of sedentary behavior during the after-school period among children aged 5-18 years

**DOI:** 10.1186/s12966-016-0419-1

**Published:** 2016-08-22

**Authors:** Lauren Arundell, Elly Fletcher, Jo Salmon, Jenny Veitch, Trina Hinkley

**Affiliations:** Institute for Physical Activity and Nutrition Research, Deakin University, 221 Burwood Highway, Burwood, VIC Australia

**Keywords:** Children, Adolescents, Sedentary behavior, After-school hours, Prevalence

## Abstract

**Background:**

Independent of physical activity levels, youth sedentary behaviors (SB) have negative health outcomes. SB prevalence estimates during discretionary periods of the day (e.g., after-school), inform the need for targeted period-specific interventions. This systematic review aimed to determine children’s and adolescents’ SB prevalence during the after-school period.

**Methods:**

A computerized search was conducted in October 2015 (analysed November 2015). Inclusion criteria were: published in a peer-reviewed English journal; participants aged 5-18 years; measured overall after-school sedentary time (ST) objectively, and/or specific after-school SBs (e.g., TV viewing) objectively or subjectively; and provided the percentage of the after-school period spent in ST/SB or duration of behavior and period to calculate this. Where possible, findings were analyzed by location (e.g., after-school care/‘other’ locations). The PRISMA guidelines were followed.

**Results:**

Twenty-nine studies were included: 24 included children (≤12 years), four assessed adolescents (>12 years) and one included both; 20 assessed ST and nine assessed SB. On average, children spent 41 % and 51 % of the after-school period in ST when at after-school care and other locations respectively. Adolescents spent 57 % of the after-school period in ST. SBs that children and adolescents perform include: TV viewing (20 % of the period), non-screen based SB (including homework; 20 %), screen-based SB (including TV viewing; 18 %), homework/academics (13 %), motorised transport (12 %), social SB (9 %), and screen-based SB (excluding TV viewing; 6 %).

**Conclusion:**

Children spent up to half of the after-school period in ST and this is higher among adolescents. A variety of screen- and non-screen based SBs are performed after school, providing key targets for interventions.

**Trial registration:**

PROSPERO registration number CRD42015010437

**Electronic supplementary material:**

The online version of this article (doi:10.1186/s12966-016-0419-1) contains supplementary material, which is available to authorized users.

## Background

Sedentary behaviors are typically performed in a sitting/lying position and result in minimal energy expenditure (≤1.5 metabolic equivalents [METS]) [1]. Evidence highlighting the negative health outcomes of sedentary behavior during childhood independent of physical activity levels is mounting [2]. For example, extensive TV viewing is positively associated with body composition and decreased academic achievement among children [2]. Many developed countries have endorsed recommendations that either place a limit on the time children should spend engaged in specific sedentary behaviors (e.g., Australia and Canada recommend less than 2 h of screen time per day [3, 4]) or recommend minimising time spent sedentary (e.g., UK guidelines recommend minimising the time spent sedentary for extended periods [5, 6]). Despite these guidelines, the majority of children exceed sedentary behavior recommendations [7–10].

One period of the day that has the potential to make a substantial contribution to children’s daily sedentary behavior levels is the after-school period. During this period, children may have more choice over the behaviors they perform compared to other times of the day, such as during school hours. Further, children perform the majority of their recreational sedentary behavior during this period [11]. For example, children perform 84 % of their daily screen-based sedentary behaviors and accrue 80 % of the daily sedentary behavior guidelines (no more than two hours per day in front of screens) during the after-school period [11]. Although defined as ‘the end-of-school to 6 pm’ [12], many studies use a variety of definitions of after-school [13–15] which makes comparing the raw minutes engaged in sedentary behavior after school difficult as longer periods provide a greater opportunity to be sedentary. Therefore, identifying the percentage of time that children and adolescents engage in sedentary behavior after school is important for informing whether this period represents a potential intervention target.

Given public health guidelines focus on limiting screen-based sedentary behaviors as well as total sedentary time, both the prevalence of engagement in specific behaviors such as TV viewing and screen-time, as well as the total time spent sedentary during this period should be examined. This literature is yet to be synthesized and reviewed systematically. Therefore, the aim of this paper was to systematically review the percentage of time children and adolescents spend during the after-school period in 1) ‘sedentary time’ defined as overall accumulated sedentary behavior measured objectively, and 2) distinct ‘sedentary behaviors’ particularly those pertinent to the guidelines (e.g., TV viewing), measured objectively or subjectively

## Methods

This review is registered with PROSPERO (registration number: CRD42015010437).

### Search procedure

A computerized search using the EBSCOhost search engine was conducted for peer-reviewed original research journal articles published in English before October 2015. The following databases were searched: Academic Search Complete, CINAHL Complete, Education Research Complete, MEDLINE, MEDLINE Complete, PsycARTICLES, Psychology and Behavioral Sciences Collection, PsycINFO and SPORTDiscus with Full Text. The key words in the search were age (“school age” OR youth OR young OR child* OR adolescen*), AND sedentary behavior (sedentar* OR television OR TV OR screen OR “electronic games” OR inactiv*), AND after-school period (after-school OR “after school” OR afternoon OR evening OR “critical window” OR “critical hours”). Reference lists of retrieved articles were also examined for potential papers. See Additional file 1: Table S1 for an example search strategy.

### Inclusion criteria

Studies were eligible for inclusion if they incorporated children aged 5-18 years and used an objective measure to assess overall after-school ‘sedentary time’ and/or used an objective or subjective measure to assess one or more individual after-school ‘sedentary behaviors’ (e.g., TV viewing, computer use). In addition, because of study variability in the duration of the after-school period, the percentage of the after-school period spent in sedentary behavior or enough information for this to be calculated also needed to be reported. That is, the paper needed to report both the duration of sedentary behavior and the length of the after-school period whereby the proportion of the after-school period spent sedentary could be calculated as follows: (duration of sedentary behavior/length of after-school period)*100. Studies were included if they examined behaviors in the afternoon once school had finished, regardless of the period start and finish times.

### Exclusion criteria

Studies examining ‘outside of school’ sedentary behavior were excluded as this often included behaviors performed before school or on weekends. Studies of special populations (e.g., overweight/obese participants or children with a disability) were excluded to allow for generalizability to the broader population. Papers examining subjective measures of overall sedentary time (i.e., the total time children were sedentary) were excluded due to the variability in survey items and sedentary behaviors examined between studies (for example, studies included a variety of combinations of TV viewing, computer use, DVD use, homework etc.). This variance in the combination of individual behaviors as contributors to overall sedentary time would have prevented accurate comparisons with the objective measures of sedentary time or with other studies using a different combination of subjectively measured sedentary behaviors to constitute overall sedentary time.

Eligibility was initially determined through a review of the title and abstract by two authors (LA and EF, inter-rater reliability of initial screening was determined by percent agreement and found to be 83 % agreement). The full-text of eligible studies were then located and reviewed.

### Data extraction and synthesis

Data for children (sample aged ≤12 years) and adolescents (sample aged > 12-18 years) were analyzed separately. Results for boys and girls are reported separately in this review if there were significant differences. All sedentary behaviors (e.g., TV viewing, motorised transport etc.) were examined to enable an exploration of the time spent in each behavior during the after-school period. The average proportion of the after-school period spent in sedentary time and each sedentary behavior was then calculated. Where accelerometry was used to assess overall sedentary time, results were separated and examined according to the accelerometer cut point used to provide a cut point-specific estimate of sedentary time. This was calculated by summing the proportion of the after-school period spent sedentary from all studies that used the cut point and dividing this by the number of those studies.

As the durations of the ‘after-school’ period varied greatly between studies (see Additional file 2: Table S2), comparisons were made via t-tests of the estimated percentage of sedentary time among studies using an after-school period of ≤ 180 min and >180 min (based on the definition of end-of-school bell time to 6 pm being approximately 180 min). No differences were observed (*p* = 0.143), therefore all studies were analysed together. This also aligns with previous findings that compared the percentage of time spent sedentary during three after-school period lengths (end-of-school to 6 pm, end-of-school to sunset and end-of-school to dinner time) and found no differences [12].

The PRISMA guidelines [16] were followed in reporting this review with the exception of conducting a methodological quality or risk of bias assessment. No methodological quality or risk of bias assessment was performed as the existing tools identified [17, 18] contained components specific to intervention or longitudinal studies. Therefore, those tools are not appropriate for prevalence reviews as previously noted in a systematic review of the prevalence of young children’s (<2 years) sedentary behavior [19]. Similarly, a previous systematic review of children’s sedentary behavior prevalence [20] did not include assessment of risk of bias.

## Results

**Fig. 1:**
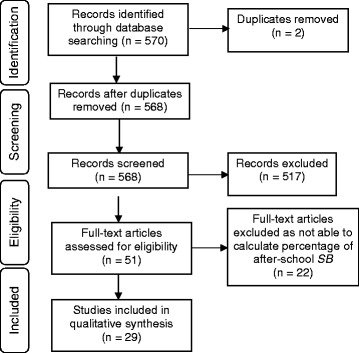
Flow chart of results from systematic search conducted in 2015

Five-hundred and seventy papers were identified, screened and assessed for their eligibility (Fig. 1) and 29 studies met the inclusion criteria. Of these, 20 assessed overall after-school sedentary time and nine assessed after-school sedentary behaviors. Among the studies assessing overall sedentary time, 16 included children (sample aged ≤12 years) [11–13, 21–33], three included adolescents (sample >12-18 years) [34–36], and one included both age groups [37]. Among the nine studies assessing sedentary behaviors, eight included children [38–45], and one included adolescents [46]. The eligible papers were published between 1996 and October 2015 and were analysed in November 2015. Study samples ranged from 20 to 2053 (mean 578) and over half of the studies (*n* = 15) had a sample of fewer than 500 participants. Study characteristics can be found in Additional file 2: Table S2.

### Country of study

Thirteen of the included studies were from the United States [22–24, 27, 29, 33, 35, 38, 39, 41, 42, 44, 45], with Australia [11, 12, 25, 43], Canada [30, 31] and the United Kingdom [13, 21, 28, 32, 37, 46] also having multiple studies. One study was identified from each of New Zealand [26], Ireland [34] and Portugal [36] and one study had a combined sample from Bulgaria, Taiwan and the United States [40].

### Child’s after school location

Among the 20 studies assessing after-school sedentary time, three assessed behavior while the children were at after-school care [22, 23, 29]. No studies examined adolescents’ behaviors while at after-school care. The remaining 17 studies either did not report where the children were after school or noted that they were at a variety of places. These studies were grouped together as at ‘other locations’ [11–13, 21, 24–28, 30–37]. No studies investigating sedentary behaviors assessed behaviors when children were at after-school care, therefore all were considered ‘other locations’ (*n* = 9) [26, 38–46].

### After-school sedentary time

#### Measurement tools

The majority of studies measured sedentary time using the ActiGraph accelerometer (*n* = 15); however, a variety of cut points were used to indicate sedentary time including <50 counts per minute (cpm) [36], <100 cpm [11–13, 23, 26, 28, 29, 32, 33, 37], < 300 cpm [30, 31], < 800 cpm [35], and <1.5METS [24]. Of the remaining five studies, one used the Actical accelerometer with a sedentary cut point of <100 cpm [25], two used direct observation (modified version of BEACHES [Behaviours of Eating and Activity for Child Health] [27], and SOFIT [System for Observing Fitness Instruction Time] [22]), one used the RT3 tri-axial accelerometer with a cutpoint of <288 cpm [21] and one used the activPAL where sedentary was defined as sitting/lying down [34].

#### Percentage of after-school sedentary time by sex

**Fig. 2:**
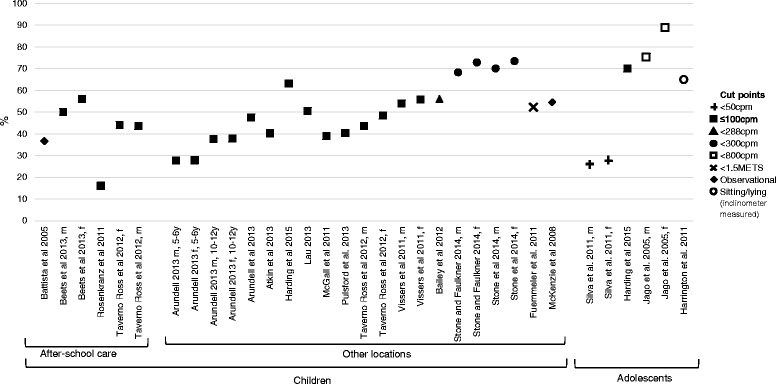
Percentage of time children (5-12 years) and adolescents (13-18 years) spend sedentary during the after-school period. Abbreviations: m = male, f = female, y = years old

Figure 2 shows the percentage of time children and adolescents spend sedentary during the after-school period by location, measure and cut point. On average, children spent 49.5 % (range 16.1 - 88.9 %) and adolescents spent 56.6 % (range 27.7 - 88.9 %) of the after-school period sedentary.

#### Percentage of after-school sedentary time by location

As shown in Fig. 2, results varied depending on the child’s location. Children attending after-school care spent on average 41.1 % (range 16.1-56.1 %) [22, 23, 29, 33], and children at other locations spent on average 50.6 % (range 27.8 %-73.5 %) [11–13, 21, 24–28, 30–33, 37] of the after-school period sedentary.

#### Percentage of after-school sedentary time by cut point

Results also varied when different cut points were used. Findings from accelerometer studies that examined sedentary time using 100 cpm showed that children spent on average 42.3 % of the after-school period sedentary [11–13, 23, 25, 26, 28, 29, 32, 33, 37], whereas the average time children spent sedentary after school when using <300 cpm as the cut point [30, 31] was 71.2 % of the period. Among adolescents, the studies that used <50 cpm [36] showed that on average adolescents spent 26.9 % of the period sedentary and in comparison the studies that used <800 cpm [35] showed adolescents spent on average 82 % of the after-school period sedentary.

### After-school sedentary behaviors

#### Measurement tools

A variety of subjective measurement tools were used to assess a range of after-school sedentary behaviors. Five studies assessed after-school TV viewing [38, 40, 43, 45, 46]. Seven studies reported screen-based sedentary behaviors and these were separated into two groups depending on whether or not their measure included TV viewing: four studies measured screen-based sedentary behaviors including TV viewing [39, 41, 42, 44] and three studies measured screen-based sedentary behaviors excluding TV viewing [40, 43, 46]. One study measured social sedentary behaviors [46], three measured homework/academics [43, 45, 46], three measured non-screen based sedentary behavior including homework/academics [41, 42, 44], one measured non-screen based sedentary behavior excuding homework/academics [43], and two measured motorised transport. [43, 46] The majority of studies used child self-report surveys asking children to report their after-school “free time” behaviors; [46] or previous day recall in 30-min blocks [41], one-hour blocks [40], in child-specific blocks (e.g., before/after child specified meal/snack) [43], or in 15 min intervals (via telephone interview) [42, 44, 45]. One study used parental proxy-report of behaviors in 15-min intervals [39] and another used observation to capture the time children spent watching TV [38].

#### Percentage of time spent in specific after-school sedentary behaviors

**Fig. 3:**
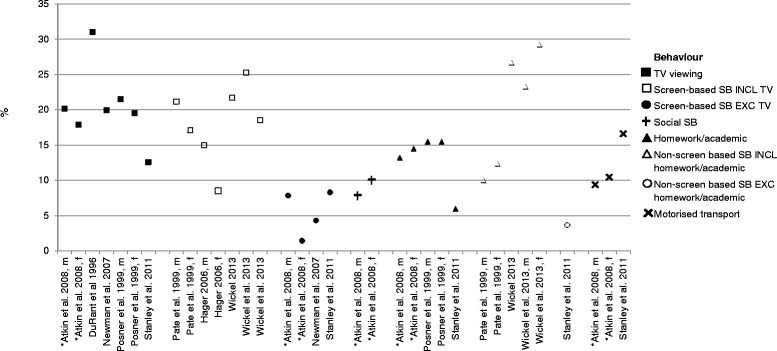
Percentage of time children and adolescents spend in specific sedentary behaviors during the after-school period. Abbreviations: m = male, f-female, *adolescent sample

Figure 3 shows the percentage of the after-school period spent in specific sedentary behaviors. As only one study reported adolescents’ after-school sedentary behaviors [46], these findings are presented alongside the children’s after-school sedentary behavior studies [38–45]. Seven findings from five studies [38, 40, 43, 45, 46] reported the percentage of the after-school period spent watching TV. TV viewing averaged 20.4 % (range 12.6 - 31 %) of the after-school period which was the highest percentage for any sedentary behavior (Fig. 3). The second largest percentage of the after-school period was spent performing non-screen based sedentary behaviors including homework (mean 20.3 %, range 10 - 29.2 %) [41, 42, 44]. This was followed by screen-based sedentary behaviors (including TV viewing;18.2 %, range 8.5 - 25.3 %) [39, 41, 42, 44], homework/academics (12.9 %, range 6 - 15.5 %) [43, 45, 46], motorised transport (12.1 %, range 9.4 - 16.6 %) [43, 46], social sedentary behaviors (adolescent boys 7.9 %, girls 10.1 %) [46], screen-based sedentary behaviors (excluding TV viewing; 5.5 %, range 1.4 - 8.3 %) [40, 43, 46], and non-screen based sedentary behaviors excluding homework/academics, such as reading, sitting quietly, writing, playing cards/puzzles/board games (3.7 %) [43].

## Discussion

This systematic review examined the prevalence of children’s and adolescents’ sedentary time and sedentary behaviors during the after-school period. The findings highlight that children spent between 41-51 % of the after-school period sedentary and that adolescents are more sedentary than children (57 %). TV viewing and other screen-based behaviors make up just 26 % or less of this period. Other non-screen based sedentary behaviors (e.g., social sedentary behaviors, motorized transport, homework, and reading) comprise 54 % of the after-school period; however, it is possible several of these behaviors occur concurrently [47, 48]. The percentage of time spent sedentary after school is greater than other periods of the day, such as recess and lunchtime, where children also have discretion over their behavior choices. For example, children aged 5-6 years and 10-12 years spend approximately 15 % and 14 % of recess sedentary respectively, and 22 % and 21 % of lunchtime sedentary respectively [49]. Further, recess and lunchtime contribute 1.8 % and 6 % of children’s daily sedentary behavior time compared to the after-school period which contributes 26 % of daily sedentary behaviors [21]. Subsequently, it would appear that the after-school period is a key period that holds great potential for interventions to target reductions in sedentary time. Further, findings suggest that interventions may need to target other specific behaviors, such as motorised transport and social sedentary behaviors, in addition to screen-based sedentary behaviors which have traditionally been targeted for change.

This review also suggests that children spent less time sedentary when in after-school care compared with ‘other locations’. This may be due to fewer sedentary pastime options (such as TV viewing and computer use) being available at after-school care. Further, after-school care may have more active structured and unstructured pastime options and facilities (e.g., active free play and organised sporting activities that use the school oval and sports equipment), and children may have more friends to be active with at after-school care compared to other locations. However, further research is needed to support this suggestion.

Findings from the included studies were highly variable. Children at after-school care are sedentary for 16.1 % to 56.1 % of the after-school period, children at other locations are sedentary for 27.8 % to 73.5 % of the period and adolescents are sedentary for 27.7 % to 88.9 % of the period. The substantial variability in estimates may be due to differences in sample sizes and accelerometer cut points. Although cut points <100 cpm may be capturing standing and light-intensity physical activity and incorrectly categorizing this as sedentary time, this threshold has been shown to most accurately represent sitting time when compared to inclinometers among 8-12 year-olds [50]. Based on findings using the cut point of 100 cpm, children spent approximately 25 min per hour of the after-school period sedentary and adolescents spent 42 min per hour of the after-school period sedentary (although this was obtained from only one study among adolescents). However, a higher sedentary cut point can greatly elevate prevalence rates. For instance, Reilly and colleagues [51] found a 321 min (5 h, 21 min) per day difference in sedentary time depending on the cut point used. This is also evident in the current review, as the prevalence of adolescents’ after-school sedentary time ranged from 27 % when using <50 cpm to 82 % when using <800 cpm. It is also important to note that there were large variations in estimates within thresholds. For example among the studies using 100 cpm, the percentage of time spent sedentary ranged from 16.1 % [29] to 56.1 % [32], highlighting the variability within the literature. This also highlights that there may be other important contextual factors impacting after-school behavior such as location (e.g., at home or after-school care) and who the children are with (e.g., alone or with friends) which require further investigation. Future research should also examine the intrapersonal, social and physical environment correlates which may further explain the variance in after-school sedentary behaviors. Such investigation would enable identification of the characteristics of children and adolescents who display high levels of sedentary time and behaviors which can subsequently be used as intervention targets. Other factors, such as sample size and characteristics, may also explain part of the variance in prevalence rates.

The most frequently measured after-school sedentary behavior was TV viewing, with children and adolescents spending approximately one fifth of the after-school period watching TV. The percentage of the after-school period spent watching TV by children and adolescents was similar (children: 21 % and adolescents 19 %) suggesting the age-related increases in after-school sedentary time observed in this review and previously observed [11] may be due to increases in participation in other sedentary behaviors (e.g., computer use or homework) [52]. However, it is hard to draw conclusions as only one study examined adolescents’ sedentary behaviors. Further, the prevalence of after-school screen-based sedentary behaviors was more than three times higher when it included TV viewing compared to when the screen-based sedentary behavior measure did not include TV. This suggests that TV viewing is the main screen-based sedentary behavior after school and a potentially important intervention target if targeting screen-based sedentary behaviors.

The prevalence of screen-based sedentary behaviors that included TV viewing was lower than the prevalence reported by studies that just reported TV viewing. This may be due to the differences between measures used as differences in recall period and question response format (e.g., behaviors during previous day, previous week etc.) and the mode of administration (e.g., phone interview or written survey) may impact on the sedentary behaviors reported [53]. The studies examining screen-based sedentary behavior including TV viewing requested participants to report their present or previous day behaviors [39, 41, 42, 44] and asked for the main behavior being performed which does not allow for reporting of concurrent sedentary behaviors. In contrast, the studies examining TV viewing used a variety of recall periods from the present day [45] to recall of behaviors on three days [40]. There may be differences in daily TV viewing behaviors that are not able to be captured via a one day recall. Alternatively, higher prevalence rates may be due to TV viewing alone being an easier behavior to recall or participants may think broadly about all screens when asked about TV viewing without realising, whereas they may be more discriminatory when asked about individual screen use. Further, participants may have been performing sedentary behaviors concurrently, however this was not assessed. Additional exploration using consistent measures would facilitate direct comparisons.

It is also important to note that approximately one-fifth of the after-school period was spent in non-screen based sedentary behaviors. Although only measured in three studies, it is possible that most of the non-screen based sedentary behaviors were homework or academic pursuits as when the measure did not include these behaviors, the prevalence was much lower (3.6 %). The similar percentage of time spent watching TV and in non-screen based sedentary behaviors suggests there may be opportunities for interventions to target sedentary behaviors other than TV viewing (e.g., through standing homework tasks [54]).

### Limitations of the current literature

The objectively-measured sedentary time findings should be interpreted with some caution as there were numerous cut points used to represent sedentary time which may influence estimations of sedentary time. Also, while few studies reported children’s behaviours while at after-school care, the majority of studies did not report where the children or adolescents were after school resulting in these studies being combined into ‘other locations’. The ‘other locations’ could include for example, a child/adolescent’s home, a friend’s or relative’s home, or the local neighbourhood and there may be important differences in sedentary time/sedentary behaviors when children and adolescents are at such locations. However, this cannot be determined from the data available. Additional studies taking into account the child’s/adolescent’s actual location after school are important for informing the development of interventions targeting those settings where children are most likely to be sedentary during the after-school period. The variability of subjective measures of sedentary behaviors also limited the ability to directly compare findings. The use of uniform survey items in future studies would assist in gaining a greater understanding of the sedentary behaviors that children and adolescents perform after school. The varying period lengths makes direct comparisons of raw minutes of sedentary behavior after school difficult. While the use of proportion of time overcomes this, the use of the standardised definition of the after-school period (end-of-school to 6 pm [12]) would further facilitate the direct comparison of future studies examining after-school behaviors. As there were no studies that examined the individual sedentary behaviors children perform during after-school care and only one that examined the individual sedentary behaviors among adolescents, the evidence in these areas is limited. Sedentary behaviors performed during after-school care (e.g., seated crafts, board games) may differ to those at ‘other’ locations; therefore, this information may assist the development of interventions targeting the after-school care setting. Lastly, a limitation is that any bias due to study methodology quality within the current review is unknown as no methodological quality or risk of bias assessment was performed; therefore, higher quality studies may show a higher or lower prevalence rate than studies of poor quality. More appropriate measures of study quality for literature reviews of prevalence studies are needed to provide estimates based on the highest quality evidence.

## Conclusion

Children and adolescents spent almost half of the after-school period sedentary with adolescents spending a greater percentage of the period sedentary than children. Few studies measured behaviors performed while at after-school care; however, the limited evidence suggests that children spent less time sedentary at after-school care than at ‘other’ locations. Children and adolescents spent the greatest percentage of the after-school period watching TV and engaged in non-screen based sedentary behaviors; however, additional research is needed that measures other sedentary behaviors (e.g., mobile phone and digital tablet use, etc.), that uses standardized survey items to enable study comparisons, and that includes the adolescent population. This review highlights children’s and adolescents’ sedentary behaviors that can be targeted for reduction though interventions in the after-school period.

## Additional files

Additional file 1: Table S1. Search strategy. Example of search strategy. (DOCX 15 kb) Additional file 2: Table S2. Study characteristics. Details of the characteristics of the studies included in this review. (DOCX 23 kb)

## Abbreviations

METS: Metabolic equivalents
Cpm: Counts per minute
BEACHES: Behaviours of eating and activity for child health
SOFIT: System for observing fitness instruction time
