# Supplementary material for: A systematic review of the prevalence of sedentary behavior during the after-school period among children aged 5-18 years
Source: Int J Behav Nutr Phys Act. 2016 Aug 22;13(1):93. doi: 10.1186/s12966-016-0419-1 (PMC4994288; doi:10.1186/s12966-016-0419-1)
Supplement: Additional file 1: Table S1. — Search strategy. Example of search strategy. (DOCX 15 kb) [file 12966_2016_419_MOESM1_ESM.docx]

Additional Table 1: Search strategy.

| 1. | school age OR youth OR young OR child* OR adolescen* |
| --- | --- |
| 2. | sedentar* OR television OR TV OR screen OR “electronic games” OR inactiv* |
| 3. | after-school OR “after school” OR afternoon OR evening OR “critical window” OR “critical hours” |
| 4. | 1 and 2 and 3 |
| 5. | limit 4 to published prior to October 2015 AND peer reviewed AND English language AND age groups: child (5-12 years) and adolescents (12-18 years) |
