# Supplementary material for: A systematic review of the prevalence of sedentary behavior during the after-school period among children aged 5-18 years
Source: Int J Behav Nutr Phys Act. 2016 Aug 22;13(1):93. doi: 10.1186/s12966-016-0419-1 (PMC4994288; doi:10.1186/s12966-016-0419-1)
Supplement: Additional file 2: Table S2. — Study characteristics. Details of the characteristics of the studies included in this review. (DOCX 23 kb) [file 12966_2016_419_MOESM2_ESM.docx]

**Additional Table 2: Study characteristics**

| **Author** | **Year** | **Sample size** | **Age in years (range or M (SD)), gender (%male)** | **Country** | **After-school period definition** | **Study design** | **Location of children** | **SB measure** | **Reported or calculated SB prevalence during the after-school period** |
| --- | --- | --- | --- | --- | --- | --- | --- | --- | --- |
| Arundell et al. | 2013 | n=2053 | Group 1: n= 608, 5-6 years, 52% male Group 2: n= 1445, 10-12 years, 45% male | Australia | End of school bell-6pm | Cohort | Not reported | Actigraph 7164, 60 sec epoch, SED<100cpm | Overall ST:  5-6yr, m: 27.8%; f: 27.9% 10-12yr, m:37.6%; f: 37.9% |
| Arundell et al. | 2013 | n=308 accelerometer data; n=108 activPAl data | 8, 42% boys | Australia | End of school bell-6pm | Cross-sectional | Not reported | Actigraph GT3X, 15 sec epoch. SB: ≤100cpm | Overall ST: 47.5% |
| Atkin et al. | 2013 | n=845, | 10.2 (±0.3), 41% boys | UK | 3-11pm | Longitudinal | Not reported | Actigraph accelerometer GT1M, SB:<100 cpm | Overall ST: 40.3% |
| Atkin et al. | 2008 | n=1,484 | 13-16 years, 38% male | UK | 3.30-6.30pm | Cross-sectional | Not reported | Self-report diary of behaviours during “free time" | TV viewing: 20.2% Screen based EXC TV: m: 7.8%; f: 1.4% Social Sedentary: m: 7.9%; f: 10.1% Homework/academics: m: 13.2%; f: 14.5% Motorised transport: m: 3.4%; f: 10.4% |
| Bailey et al. | 2012 | n=135 | 11.7 (±1.4), 42% male | UK | End of school bell - 6.30pm | Baseline data from PA (intervention) | Not reported | RT3 triaxial accelerometers, 60 sec epoch, SED: <288 cpm | Overall ST: 56.1% |
| Battista et al. | 2005 | n=533 | grade 4-6, 50% male | US | 2-6pm | Cohort | After-school care | SOFIT (System for Observing Fitness Instruction Time) | Overall ST: 36.7% |
| Beets et al. | 2013 | n=785 | Kinder to year 5, 50% male | USA | Average of 208 minutes/day (range 60-240) | Observational | After-school care | Actigraph GT1M, 5sec epoch, SED <100cpm | Overall ST: m: 50.1%; f: 56.1% |
| DuRant et al. | 1996 | n=138 | 5-6 years %male not reported | USA | >150mins after 3pm​ | Cross-sectional data from longitudinal study | Not reported | Observational: Children's activity rating Scale (CARS) | TV viewing: 31% |
| Fuemmeler et al. | 2011 | n=45 parent-child triads (mums, dads and children) | Child age: 10.6 (±0.7) 51% male. Mother age 40.6 (±5.6), father age: 42.8 (±6.2) | USA | 3-7pm | Cross-sectional | Not reported | Actigraph 7164, 60sec epoch, SED<1.5METs | Overall ST: 52.3% |
| Harding et al. | 2015 | n= 363 | 12.0 (±0.4), 39% male, followed to 15.4 yrs (±0.5), 39% male, | UK | End of school bell - 9pm | Cohort | Not reported | Actigraph accelerometer GT1M, SED<100cpm | Overall ST: 12yr: 63.2%; 15yr: 70.1% |
| Harrington et al. | 2011 | n=102 | 15-18 years (0% male (female sample) | Ireland | 4-10pm | Cross-sectional | Not reported | activPAL inclinometers | Overall ST: 65% |
| Hager | 2006 | n=80 | 10 (±1.0), 50% male | USA | 5.30-6pm | Cross-sectional | Not reported | Parent report TV and computer/ video game use | Screen based INCL TV: m: 15%; f: 8.5% |
| Jago et al. | 2005 | n= 81 | 13.3 (±0.5), 54% male | USA | 3pm-6.59pm | Cross-sectional | Not reported | Actigraph MTI SED<800cpm, and self-report previous day electronic recreation use | Overall ST: m: 75.3%; f: 88.9% |
| Lau | 2013 | n=20 parent-child diads, | 6.25 (±0.64), 55% male | Australia | 3.30-7pm | Cross-sectional from cohort study | Not reported | Actical acceleromter, 15sec epoch, SED<100cpm | Overall ST: 50.5% |
| McGall et al. | 2011 | n=60, | 8.3years (±0.7), 54% male | New Zealand | 3-5pm | Cross-sectional | Not reported | actigraph GT1M, 60-sec epoch, sed <100 cpm, valid data 4+ days. Treuth et al (Med Sci Cports Exerc 2004: 36: 1259-66) | Overall ST: 39% |
| McKenzie et al. | 2008 | n=139 | 6.5 (±0.98), 50% male | USA | 2 x 30 minute periods between end of school and dinner | Cross-sectional | Home | Observational: modified version of BEACHES (Behaviours of Eating and Activity for Child Health: Evaluation System). | Overall ST: 54.6% |
| Newman et al. | 2007 | n=742 | 10.13, 48% male | Bulgaria, Taiwan and the USA | End of school until 10pm | Cross-sectional | Not reported | Self-report | Screen based EXC TV: 4.48% |
| Pate et al. | 1999 | n= 181 | 10.7 (±0.7), 46.3% male. n=82 at follow-up | USA | 3pm-11.30pm | Longitudinal | Not reported | Self-report Previous Day PA recall (PDPAR). | Screen based INCL TV: m: 21.2%; f: 17.1% Non-screen SB INCL homework/academics: m: 10%; f: 12.4% |
| Posner et al. | 1999 | n=194 (at 2-year follow-up) | 9.1 (±0.5), 54% boys | USA | 2.45-6pm or 3.35-6.35pm (dependant on dismissal time) | Cohort | Variety | Self-report | Homework/academics: 15.5% |
| Pulsford et al. | 2013 | n= 629 | 10.95 (±0.4), 51% male | United Kingdom | 3-11pm | Cross-sectional | Not reported | Actigraph GT1M (Wrist worn), 10sec epoch, SED<100cpm | Overall ST: 40.4% |
| Rosenkranz et al. | 2011 | n=240 | 9.3 (±0.7), 51% male | USA | Not reported | Cross-sectional | After-school care | Actigraph GT1M, 30sec epoch, SED<100cpm | Overall ST: 16.1% |
| Silva et al. | 2011 | n=24 | 11.04 (±1.45), 50% male | Portugal | 6.01pm-8pm | Cross-sectional | Not reported | Actigraph, 7164, 60sec epoch, SED<50cpm | Overall ST: m: 26.1%; f: 27.7% |
| Stanley et al. | 2011 | n=794 | 11.9 (±1.6), 48% male | Australia | 90 minutes after end of school bell | Cross-sectional | Not reported | Self-report 1 day recall using Multimedia Activity Recall for Children and Adults (MARCA). | Screen based EXC TV: 8.3% Homework/academics: 6% Non-screen SB EXC homework/academics: 3.7% Motorised Transport: 16.6% |
| Stone et al. | 2014 | n=856 | 11 (±0.6), 45% males | Canada | 2hrs after school bell | Cohort | Not reported | Actigraph GT1M, 5sec epoch, SED<300cpm | Overall ST: m: 70.1%; f: 73.5% |
| Stone and Faulkner | 2014 | n=856 | 11 (±0.6), 45% males | Canada | 2hrs after school bell | Cohort | Not reported | Actigraph GT1M, 5sec epoch, SED<300cpm | Overall ST: m: 68.3%; f: 72.9% |
| Taverno Ross et al. | 2012 | n=662 | 10.6 (±0.5), 45% male | USA | after school bell - 6pm | Cross-sectional | Home or After-school Care | Actigraph GT1M and GT3Xm, 60sec epoch, SED<100cpm | Overall ST (home): m: 43.6%; f: 48.4% Overall ST (after-school care): m: 43.6%; f: 44.1% |
| Vissers et al. | 2011 | n=1697 | 10.3 (±0.3), 44% males | England | 12noon - 9pm | Cross-sectional | Not reported | Actigraph GT1M, 5sec epoch, SED<100cpm | Overall ST: m: 53.9%; f: 55.8% |
| Wickel. | 2013 | n=862 year 3 students and 954 year 4 students | 10, 50% male | USA | 3-6pm | Cohort | Variety | Self-report present day screen- and non-screen based SB recall | Screen based INCL TV: 21.7% Non-screen SB INCL homework/academics: 26.7% |
| Wickel et al. | 2013 | n=886 | 9-11, 50% male | USA | 3-6pm | Cohort | Variety | Self-report present day screen- and non-screen based SB recall | Screen based INCL TV: m: 25.3%; f: 18.5% Non-screen SB INCL homework/academics: m: 23.3%; f: 29.2% |
